# Usefulness of digital infrared thermography video using the FLIR T560 in detecting hypothermia associated with lumbosacral radiculopathy following spinal stenosis: A CARE-compliant case report

**DOI:** 10.1097/MD.0000000000041874

**Published:** 2025-03-14

**Authors:** Yewon Jang, Sungho Kim, Min Cheol Chang

**Affiliations:** a Department of Electronic Engineering, Yeungnam University, Gyeongsan-si, Republic of Korea; b Department of Physical Medicine and Rehabilitation, College of Medicine, Yeungnam University, Daegu, Republic of Korea.

**Keywords:** hypothermia, radiculopathy, sensor, spinal stenosis, temperature

## Abstract

**Rationale:**

Here, we investigated the effectiveness of the FLIR T560 thermal imaging camera in detecting hypothermic changes associated with radiculopathy caused by spinal stenosis. This study aimed to determine whether the FLIR T560 could serve as a portable, efficient tool for diagnosing radiculopathy and its related sympathetic dysfunction in clinical practice.

**Patient concerns:**

A 77-year-old male had a 1-year history of neuropathic pain in the right distal lower leg, confirmed as L5 and S1 radiculopathy due to central stenosis at L4–5 and L5–S1, as shown by MRI and electrodiagnostic studies. The FLIR T560 recorded thermal images in an insulated room, revealing a significantly lower surface temperature (approximately 1.0°C difference) on the lateral and posterior right distal lower leg and right toes compared with the left side. By referencing the color map and scale, we estimated that the relative temperature difference between corresponding areas was approximately 1.0°C.

**Diagnoses:**

L5 and S1 radiculopathy due to central stenosis at L4–5 and L5–S1.

**Interventions:**

A transforaminal epidural steroid injection targeting the right L5 and S1 nerve roots was conducted.

**Outcomes:**

The patient’s pain improved significantly.

**Lessons:**

The FLIR T560 camera offers a portable and convenient alternative to traditional digital infrared thermographic imaging, allowing real-time thermal imaging in a clinical setting without the need for additional equipment. Our case report suggests that the FLIR T560 is a valuable tool for detecting sympathetic dysfunction associated with radiculopathy. Further studies with larger patient populations are recommended to validate its clinical utility.

## 1. Introduction

Compression or inflammation of the spinal nerve roots due to a herniated disc or spinal stenosis causes radiculopathy, which manifests as sensory (pain, hypesthesia, or hypoalgesia) and motor (paresis) symptoms.^[1]^ In addition to these sensory and motor symptoms, compression or inflammation of the spinal nerve root can also cause hypothermia in areas innervated by the affected nerve root.^[2,3]^ This is likely due to dysfunctional sympathetic fibers that exit the spinal foramen alongside the motor fibers, which supply the skin vessels and sweat glands in those areas.^[4–6]^ Numerous studies have reported sympathetic dysfunction following radiculopathy.^[4–7]^ Sympathetic excitation results in vasoconstriction in the area innervated by the corresponding nerve root and stimulates sweat secretion from the sweat glands, leading to a decrease in temperature.^[4–7]^

Based on this concept, digital infrared thermographic imaging (DITI) has been used to visualize changes in skin temperature, primarily hypothermia, in patients with radiculopathy.^[3,4]^ Reports indicate that DITI is useful for confirming the diagnosis of radiculopathy and differentiating between affected nerve roots. However, most DITI systems require a plugged-in power source, and the entire setup, including the camera, motorized stand, and console unit, is large and heavy. Consequently, significant space is needed to set up the equipment and perform the imaging. Also, to capture patient temperature imaging data using DITI, patients must move to the imaging room and assume a fixed position and posture.^[8]^

To address these limitations, we utilized the FLIR T560 (FLIR, Seoul, Republic of Korea): a high-performance thermal imaging camera designed for scientific applications that captures the thermal infrared band in a manner similar to DITI.^[9,10]^ Unlike DITI, which requires a personal computer, the FLIR T560 integrates an integrated camera display, allowing for video review without additional equipment. This portable, lightweight, battery-operated camera enables immediate patient temperature recordings in the examination room and can be maneuvered to accommodate various patient postures. Table 1 compares the specifications of the IRIS-XP (Medicore, Hanam-si, Republic of Korea), a DITI model commonly used in clinical settings, with our thermal infrared camera, the FLIR T560.^[9,10]^

**Table 1:** Specifications for IRIS-XP (digital infrared thermographic imaging) and FLIR T560.

	IRIS-XP	FLIR T560
IR image resolution (pixels)	384 × 288 (640 × 480 is optional)	640 × 480
Frame rate (Hz)	9 or 30	30
Spectral range (μm)	8–14	7.5–14
Temperature measurement range (°C)	14.5~40	−20~120
Temperature sensitivity (°C)	0.05	0.04
Size (mm)	Camera + motorized stand: 460 × 550 × 155~1420 Console unit: 600 × 800 × 1330	Camera only: 140 × 201 × 167
Power supply method	Connect to a power outlet	Use battery
Compensation function of room temperature	O	O
Analytics software provided	O	O
PC system included	O	X
Portability	X	O

IR = infrared, PC = personal computer.

In this study, we report a case in which hypothermia associated with lumbosacral radiculopathy following lumbar spinal stenosis was detected using the FLIR T560.

## 2. Case report

A 77-year-old man with no significant previous medical history aside from hypertension presented with neuropathic pain (tingling and paresthesia) on the lateral and posterior sides of his right distal lower leg, which had persisted for 1 year. His numeric rating scale score was 7 (range: 0–10, 0: no pain, 10: the worst pain imaginable) and his pain was aggravated by walking and standing. Physical examination revealed hypoalgesia in the right L5–S1 dermatome, and weakness in the right extensor hallucis longus muscle (Manual Muscle Test grade: 4).

**Figure 1.**
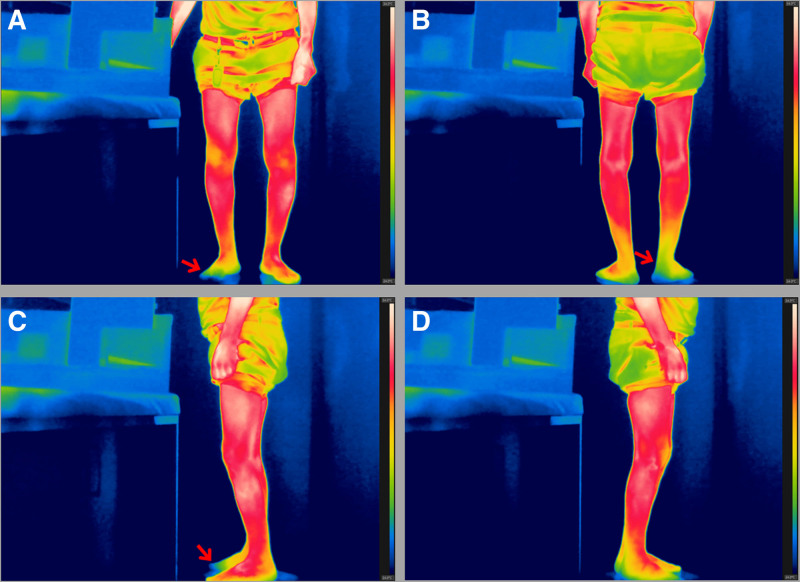
A 77-year-old man with pain in the lateral and posterior sides of the right distal lower leg due to spinal stenosis on L4–5 and L5–S1. The image reveals blue and dark colors (red arrows) at the lateral and posterior sides of the right distal lower leg and right toes. (A) Front, (B) back, (C) left lateral, and (D) right lateral sides.

Bilateral deep tendon ankle reflexes were hypoactive, while knee reflexes were normal. Ankle clonus was not assessed in either ankle. Lumbar magnetic resonance imaging (MRI) conducted 4 months previously at a local clinic revealed central stenosis at L4–5 and L5–S1 (Fig. 1). An electrodiagnostic study showed right L5 and S1 radiculopathy.

The FLIR T560, an infrared camera operating in the LWIR band (7.5–14 μm), was used to capture videos of the patient’s body temperature distribution. The FLIR T560 can detect temperature differences as small as 0.04°C, with a measurable range from −20 to 120°C, ±2°C.^[9]^ To minimize temperature fluctuations, the experiment was conducted in an insulated room with 2 experimenters and the patient.^[11]^ During the recording, the patient stood approximately 3 m from the camera and rotated their body to provide optimal views of the suspected areas of temperature variation.

For accurate infrared imaging, calibration parameters were set at 25°C room temperature, 50% humidity, 3 m recording distance, 0.98 skin surface emissivity, and 25°C surface reflectance temperature.^[8,11,12]^ The frames from the thermal infrared video are shown in Figure 1 (see Video S1, Supplemental Digital Content, http://links.lww.com/MD/O519), which demonstrates the video captured using the FLIR T560.

The video was captured with a color mapping range of 24 to 34°C, ensuring consistent temperature-to-color mapping across all frames. According to the color bar displayed on the right side of each image, red and brighter colors indicate higher surface temperatures, while blue and darker colors represent lower temperatures.

The temperature distribution revealed that the lateral and posterior sides of the right distal lower leg and right toes exhibited significantly lower surface temperatures when compared with the left. By referencing the color map and bar, we estimated that the relative temperature difference between corresponding areas was approximately 1.0°C.

Considering the pain experienced by the patient and the results of the imaging and electrodiagnostic studies, we determined that the patient’s pain was induced by radiculopathy affecting the right-sided L5 and S1 due to central stenosis at L4–5 and L5–S1. For pain management, we performed a transforaminal epidural steroid injection on the right L5 and S1 nerve roots. At both the 1-month and 2-month follow-ups, the patient reported a significant reduction in pain (numeric rating scale: 1) in the right distal lower leg.

Written informed consent was obtained from the patient for the publication of this case report and the accompanying images. Institutional approval was required to publish the case details, and our study was approved by the institutional review board of Yeungnam University Hospital.

## 3. Discussion

The patient in our case study experienced pain on the lateral and posterior sides of the right distal lower leg, which was caused by spinal stenosis at L4–5 and L5–S1. The FLIR T560 images showed blue and dark colors in the corresponding areas, including the right toes, indicating a reduction in skin temperature. These color changes were attributed to the deterioration of the sympathetic nerves because of right-sided L5 and S1 radiculopathy caused by spinal stenosis on L4–5 and L5–S1, a diagnosis that was confirmed through electrodiagnostic tests and the patient’s positive therapeutic response to transforaminal epidural steroid injections targeting the right L5 and S1 nerve roots.

According to Planck’s law, all objects with a surface temperature above absolute zero (0°K) emit electromagnetic radiation.^[8,13,14]^ Infrared cameras detect and visualize this radiation in the thermal infrared band.^[13]^ The intensity of this radiation is proportional to temperature, and temperature differences are represented using a user-selected color map, facilitating easy visualization and analysis of temperature distribution.^[14]^ This operating principle is similar to that of traditional DITI. However, the FLIR T560 offers greater portability compared to DITI as it features a direct image display on the camera itself, eliminating the need for a PC or separate monitor. This convenience allows for the immediate capture, in the clinic, of images from suspected areas, enabling rapid image analysis and prompt diagnosis.

By using the FLIR T560, pain physicians can place the device beside them while examining a patient, allowing for real-time measurement of the patient’s skin temperature. This capability facilitates the approximate identification of nerve lesions involving sympathetic dysfunction, such as radiculopathy and complex regional pain syndrome, and helps differentiate which nerves are affected. Additionally, since it captures video rather than just images, the FLIR T560 offers the advantage of real-time temperature measurements across all areas of the body, not just specific regions.

In conclusion, the FLIR T560 thermal imaging camera revealed hypothermia in the painful regions on the lateral and posterior side of the patient’s right distal lower leg, which was associated with L5 and S1 radiculopathy caused by spinal stenosis at L4–5 and L5–S1. We believe that the FLIR T560 can be a valuable tool for pain physicians, offering greater portability and convenience compared with traditional DITI systems. The FLIR T560 enables real-time measurement of skin temperature in a clinical setting, allowing for the rapid identification of nerve lesions involving sympathetic dysfunction. Additionally, the FLIR T560’s ability to display images on the device itself simplifies the diagnostic process by eliminating the need for additional equipment, such as personal computers or monitors. Our study has some limitations. First, the findings in our study are based on a single case, limiting the generalizability of the results. Second, our study did not compare the results of the FLIR T560 with other diagnostic imaging techniques, such as DITI or 3-phase bone scan, to assess its relative effectiveness in diagnosing radiculopathy and associated hypothermia. Third, we did not provide quantitative measurements of temperature differences, which could have offered a more precise assessment of the temperature differences between the affected and unaffected areas. Further studies compensating these limitations are warranted in the future.

## Author contributions

**Conceptualization:** Yewon Jang, Sungho Kim, Min Cheol Chang.

**Data curation:** Yewon Jang, Sungho Kim, Min Cheol Chang.

**Formal analysis:** Yewon Jang, Sungho Kim, Min Cheol Chang.

**Funding acquisition:** Sungho Kim, Min Cheol Chang.

**Investigation:** Yewon Jang, Sungho Kim, Min Cheol Chang.

**Methodology:** Yewon Jang, Sungho Kim, Min Cheol Chang.

**Project administration:** Yewon Jang, Sungho Kim, Min Cheol Chang.

**Resources:** Sungho Kim, Min Cheol Chang.

**Software:** Yewon Jang, Sungho Kim, Min Cheol Chang.

**Supervision:** Min Cheol Chang.

**Validation:** Yewon Jang, Sungho Kim, Min Cheol Chang.

**Visualization:** Yewon Jang, Sungho Kim, Min Cheol Chang.

**Writing – original draft:** Yewon Jang, Sungho Kim, Min Cheol Chang.

**Writing – review & editing:** Yewon Jang, Sungho Kim, Min Cheol Chang.

## Supplementary Material


